# Altitudinal gradients of soil and vegetation carbon and nitrogen in a high altitude nature reserve of Karakoram ranges

**DOI:** 10.1186/s40064-016-1935-9

**Published:** 2016-03-12

**Authors:** Arshad Ali Shedayi, Ming Xu, Iqnaa Naseer, Babar Khan

**Affiliations:** Key Laboratory of Ecosystem Network Observation and Modeling, Institute of Geographic Sciences and Natural Resources Research, University of Chinese Academy of Sciences, Beijing, China; Department of Biological Sciences, Karakoram International University, Gilgit, Pakistan; Department of Ecology, Evolution and Natural Resources, School of Environmental and Biological Sciences, Rutgers University, New Brunswick, NJ USA; Department of Botany, University of Gujrat, Gujrat city, Pakistan; Gilgit conservation and information centre, WWF, Gilgit, Pakistan

**Keywords:** Soil organic carbon, Soil inorganic carbon, Soil total nitrogen, Soil total carbon, Leaf total carbon, Leaf total nitrogen, Altitude, Forest type, Litter mass, Herbaceous biomass

## Abstract

This study aimed to investigate the correlation of carbon and nitrogen in soil and leaves with the altitude, vegetation type, herbaceous biomass (HB), litter mass (LM) and with each other. Soil and leaf samples collected from different forest types along altitudinal gradients in the Karakoram Mountains. Dry and gas law methods were used for the chemical analysis. Regression models used for correlation analysis and T test for comparison. The correlation of soil total carbon (STC) and soil total nitrogen (STN) along altitudinal gradients and correlation between soil organic carbon (SOC) and STN was significantly positive with the values R^2^ = 0.1684, p = 0.01, R^2^ = 0.1537, p = 0.009 and R^2^ = 0.856, p = 7.31E−10 respectively, while it was non-significant between soil inorganic carbon (SIC) and altitude and also between SIC and STN. The concentration of SOC and STN was highest in the broad leaved *Betula utilis* forest (22.31, 1.6 %) and least in the mixed (Pinus, Juniper, Betula) forest soil (0.85, 0.09 %) respectively. In the tree species leaf total carbon (LTC) and leaf total nitrogen (LTN) were highest in the *Pinus wallichiana* (PW) (632.54, 19.77), and least in the *Populus alba* (87.59, 4.06). In the shrub species LTC and LTN nitrogen were highest in the *Rosa webiana* (235.64, 7.45) and least in the *Astragalus gilgitensis* (43.45, 1.60) respectively. Total carbon and total nitrogen showed a slightly decreasing and increasing trend with altitude in the leaf and soil samples, respectively. The mean nitrogen and carbon was higher in the leaves of trees (3, 97.95) than in the shrubs (2.725, 74.24) and conifers (2.26, 76.46) than in the leaves of the deciduous (2, 46.36) trees. The correlation between LTC and STN was non-significant. Strong significant (R^2^ = 0.608, p = 0.003) and weak non-significant (R^2^ = 0.04, p = 0.32) relationships were found in STN and STC with LM and HB respectively. SOC (75.15 %) was found to be the main contributor to the STC (76.20 %) as compared to the SIC (1.05 %). Furthermore, SOC (75.15 %) was the major constituent to the soil organic matter (SOM) as compared to the STN (5.85 %). The vegetation type and litter both showed high impact on STN and STC, while altitude and HB showed very little or no impact on carbon and nitrogen concentrations. However, altitude puts great impact on shaping vegetation structure due to decreasing temperature along the elevation and other climatic factors which are further responsible in controlling the carbon and nitrogen concentration.

**Electronic supplementary material:**

The online version of this article (doi:10.1186/s40064-016-1935-9) contains supplementary material, which is available to authorized users.

## Background

Carbon and nitrogen are the two chemical elements in organic matter which are the most important, especially in their relation and proportion to each other. Carbon and nitrogen are both important for energy generation and growth regulation (Miller 2000) and both play important roles in global warming and climate change (IPCC 2007). According to Ajani (2011) and Ajani et al. (2013) there are three important carbon reservoirs for global carbon cycle such as (1) primary (geocarbon and biocarbon), (2) anthropogenic (stockpiles, processed), (3) atmosphere and ocean (atmosphere and ocean water). Soil organic carbon (SOC) is extremely important in the global carbon cycle. Carbon sequestration in non-disturbed ecosystems are the best sinks of carbon and mitigate global climate change (Parras-Alcántara et al. 2015). The altitudinal and topographic variation play important role in the SOC distribution as the SOC content from the topsoil varied largely ranging between 27.3 and 39.9 g kg^−1^ in a national park of the southern Spain along altitudinal gradients (Parras-Alcántara et al. 2015). Deforestations is the second largest cause of greenhouse gases after fossil fuel (van der Werf et al. 2009). Cutting of forest trees to produce goods or heat release the carbon about million tons of carbon into the atmosphere per year (FAO 2010). Forest and wetland destruction is the main source of global climate change (Erwin 2009; Riegel et al. 2013). Nitrogen in soil exists in many forms and easy transfer from one to another form. Nitrogen process is biologically influenced and which is further influenced by the climatic conditions, physical and chemical properties of the soil. Interactions between the terrestrial nitrogen (N) and carbon (C) cycles shape the response of ecosystems to global change (Zaehle 2013). Nitrous oxide also contributes to stratospheric ozone layer depletion (Ravishankara et al. 2009) and has more global warming potential than CO_2_ (IPCC 2007; Nguyen et al. 2014). The concentration of N_2_O in the atmosphere is increasing by 0.8 % annually (IPCC 1994). The concentration of CO_2_ has increased from 280 ppm in preindustrial times to 392 ppm in the early twenty-first century (Tans 2012), highlighting that a reduction in the concentration of both gases would aid in the mitigation of climate change (Nguyen et al. 2014). Nitrous oxide is produced from nitrification and denitrification processes (Davidson et al. 1986) and are influenced by pH (Law et al. 2011), soil moisture, and availability of C and N substrates (Beare et al. 2009; Nguyen et al. 2014).

Carbon accumulation depends on nitrogen accumulation and nitrogen accumulation depends on the conversion of atmospheric nitrogen into nitrate by legumes. The vegetation type and forest composition influences the accumulation of carbon and nitrogen. For example, leguminous plant species and C4 plants increase the soil carbon and nitrogen concentration while the C3 plant species decrease the soil carbon and nitrogen concentration (Knops and Tilman 2000). Soil plays an important function in retaining soil nitrogen (N) (Vesterdal et al. 2008). The knowledge about the effects of tree species on soil carbon is important for the mitigation of greenhouse gases and recently gained much significance (Jandl et al. 2007). Studies show the influence of tree species on C and N cycling (Menyailo et al. 2002). Tree species are one of several factors that influence soil carbon and nitrogen inputs and outputs and soil C and N are determined by differences in inputs and outputs in soil. Comparative studies of tree species growing under different conditions can be beneficial for checking their influence (Binkley 1995). Tree species further depend on the differences in soil conditions such as parent material or land use (Vesterdal et al. 2008). Tree species influence is often first detectable in forest floors whereas mineral soil differences emerge later (Vesterdal et al. 2002). Soil disturbances and land use changes are responsible for releasing soil carbon into the atmosphere (Cochran and Collins 2007). Tree species composition determines the variability in soil C/N ratios and N retention (Lovett et al. 2002). Deciduous and coniferous species have large variability in forest floor C and N (Ovington 1954). Deciduous forests with large forest floor C pools store less carbon in soil (Oostra et al. 2006), while more carbon has been reported in soils under spruce and beech in the central and western parts of Europe (Berger et al. 2002).

Carbon and nitrogen both are local indicators of global climate change (Zaehle 2013). We can predict future global climate change based on knowledge of the carbon accumulation in any ecosystem. We conducted this study at a high altitude mountain nature reserve. Mountain ecosystems play important role as being the local source and sink of carbon and nitrogen for global climate change. The effects of anthropogenic activities and changing climatic conditions have severe impacts on the mountain forest ecosystem; this may result in the release of carbon and nitrogen stocks along with creating other serious local consequences. The altitudinal gradient plays vital role in the distribution of SOC, it is therefore suggested to include elevation in SOC models to estimate at local and regional level (Parras-Alcántara et al. 2015).

Understanding the distribution of organic/inorganic carbon storage in soil profile is crucial for assessing regional, continental and global soil C stores and predicting the consequences of global change (Wang et al. 2010). Soil is considered to be the most important sink of greenhouse gases. The topsoil is the component of the soil system showing most rapid responses to environmental changes, such as alterations in temperature, precipitation and nitrogen deposition (Liao et al. 2009). Changes in the topsoil are particularly important for exploring ecosystem response and functioning (Franzluebbers and Stuedemann 2010).

The response of soil carbon to the global change is important to find both soil organic carbon (SOC) and soil inorganic carbon (SIC), but many studies have focused on SOC and less attention drawn on SIC (Mi et al. 2008; Shi et al. 2012). However, the proportions of carbonates in soil total carbon are usually small (Chatterjee et al. 2009), thus the method without the direct measurement of SIC could produce a large relative error. Therefore, studies focused on SIC based on measured data help us to reduce the uncertainties of previous studies and to predict response of soil carbon to global changes (Shi et al. 2012). SIC pools exchange C with the atmosphere through a series of physical and chemical reactions, such as C sequestration by carbonate formation or CO_2_ release by acidification and leaching (Ouyang et al. 2008: Shi et al. 2012). Terrestrial biospheres are the largest carbon pools, a small change in soil carbon cause a significant alteration of atmospheric CO_2_ concentration (Trumbore and Czimczik 2008; Shi et al. 2012). Therefore, both SIC and SOC pools should be considered in order to more accurately predict future soil carbon dynamics (Shi et al. 2012).

This is the first comprehensive study of its kind in this very important alpine nature reserve. The whole region depends on this nature reserve for food, fuel, timber, water, electricity and livestock along with other recreational and biodiversity related benefits. This study aims to find relationships between carbon and nitrogen in soil, vegetation, HB and LM along altitudinal gradients and forest types. We also aimed to determine the relationship of the parameters with each other for example TC, TN, OC and IC in soil and TC and TN in leaves. This study will help researchers foster further in depth investigations and provide a clearer picture of the impact and relationship of altitude, forest type, herbaceous biomass and litter mass on carbon and nitrogen concentrations and will help predict future carbon and nitrogen accumulation in alpine forests.

## Results

**Fig. 1:**
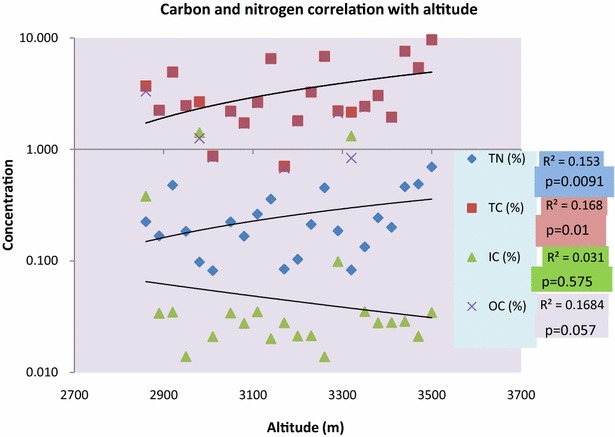
STC, SOC, STN and SIC correlation with altitude

**Fig. 2:**
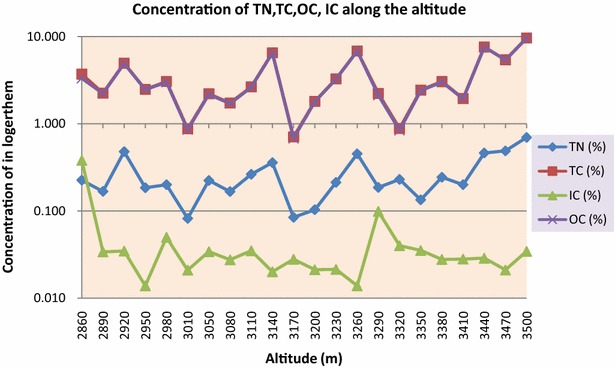
Concentration of STN, STC, SIC and SOC along altitudinal gradients

**Fig. 3:**
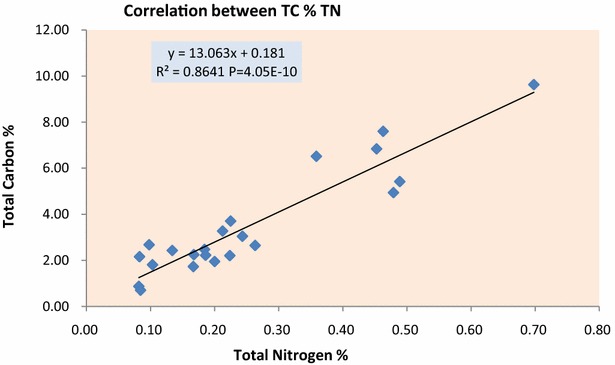
Soil: correlation between STN and STN

**Fig. 4:**
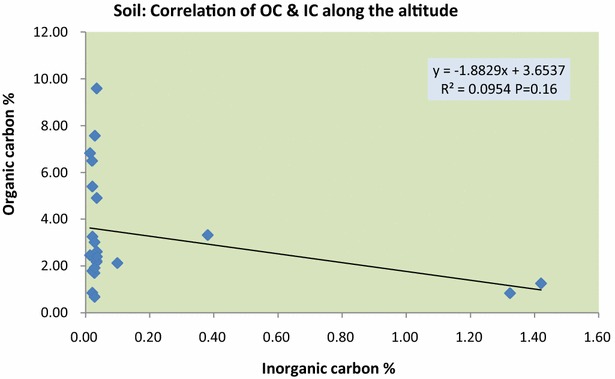
Soil: correlation between SOC and SIC

**Fig. 5:**
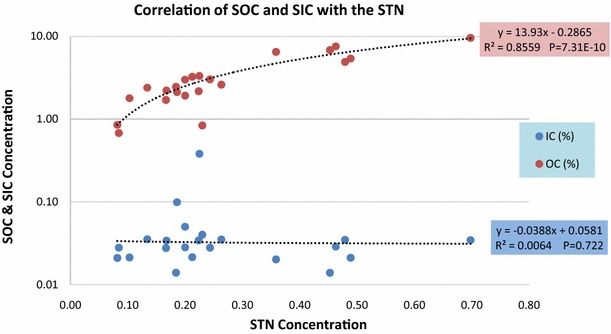
Soil: correlation of SOC and SIC with STN

The trend of soil total carbon (STC), soil total nitrogen (STN) and soil organic carbon (SOC) showed significant correlation with altitude as p > 0.01 while soil inorganic carbon (SIC) showed a nonsignificant negative correlation with altitude as p = 0.575 (Fig. 1; Table 1). Over all soil total carbon (STC) was found to be highest along the altitudinal gradients 76 % followed by soil organic carbon (SOC) 75.15 %, soil total nitrogen (STN) 5.85 % and soil inorganic carbon (SIC) 1.05 %. Highest STC and SOC was found to be 9.63 and 9.59 % at altitude 3500 m each, while lowest was 0.71 and 0.68 % at 3170 m each. Highest SIC was found to be 0.38 % at altitudes 2860 m while lowest was 0.01 % at 2950 m and 3260 m each. Highest STN was found to be 0.70, 0.49 and 0.48 % at 3500, 3440 and 2920 m respectively while lowest was 0.08 % at altitudes 3010 and 3170 m each (Fig. 2; Additional file 1: Annexure S1). Nitrogen and carbon showed a strong positive correlation with altitude with R^2^ = 0.864 and differ significantly at p = 4.05E−10. Inorganic carbon and organic carbon showed a weak negative correlation and did not differ significantly at R^2^ = 0.095 and p = 0.16 (Figs. 3, 4). There was a strong significant positive correlation found between the SOC and STN, while there was a non-significant correlation observed between SIC and STN (Fig. 5).

**Table 1:** Concentration (%) of STN, STC, SIC and SOC along the altitude

Altitude (m)	Dominant trees	STN (%)	STC (%)	SIC (%)	SOC (%)
2860	*Picea smithiana*	0.23	3.70	0.38	3.32
2890	*Pinus wallichiana*	0.17	2.25	0.03	2.21
2920	*Pinus wallichiana*	0.48	4.94	0.03	4.91
2950	*Picea smithiana*	0.18	2.48	0.01	2.46
2980	*Pinus wallichiana*	0.20	3.05	0.05	3.00
3010	*Pinus wallichiana* + *Juniperus excelsa*	0.08	0.87	0.02	0.85
3050	*Juniperus excelsa* + *Astragalus*	0.22	2.21	0.03	2.17
3080	*Juniperus excelsa*	0.17	1.73	0.03	1.70
3110	*Juniperus excelsa* +*Populus*	0.26	2.65	0.04	2.61
3140	*Picea smithiana*	0.36	6.52	0.02	6.50
3170	*Juniperus excelsa*	0.08	0.71	0.03	0.68
3200	*Pinus wallichiana*	0.10	1.81	0.02	1.79
3230	*Pinus wallichiana*	0.21	3.27	0.02	3.25
3260	*Picea smithiana*	0.45	6.84	0.01	6.83
3290	*Betula utilis*	0.19	2.22	0.10	2.13
3320	*Pinus wallichiana*	0.23	0.88	0.04	0.84
3350	*Pinus wallichiana*	0.13	2.43	0.04	2.40
3380	*Betula utilis*	0.24	3.05	0.03	3.02
3410	*Juniperus excelsa*	0.20	1.95	0.03	1.92
3440	*Betula utilis*	0.49	7.60	0.03	7.57
3470	No tree	0.46	5.42	0.02	5.40
3500	*Betula utilis*	0.70	9.63	0.03	9.59
	Total	5.85	76.20	1.05	75.15

**Fig. 6:**
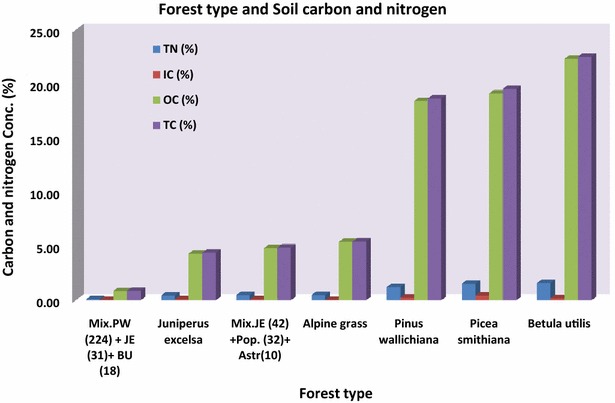
Forest type and STC, SOC, SIC and STN

Soil total carbon (STC), soil organic carbon (SOC) and soil total nitrogen (STN) was highest in the broad leaved *Betula utilis* (BU) forests, followed by *Picea smiothiana* (PS), *Pinus willachina* (PW), alpine grass, mixed forests (JE, Pop, Astr), *Juniperus excelsa* (JE) and in the Mixed forest (PW, JE, BU). It showed soil organic matter (SOM) was in high concentration in the broad leaved forest as compared to the mixed forest and Juniper forest. Broad leaved forest soil is rich with carbon and nitrogen and contributes the most as carbon sinks in the area. Highest STC, SOC and STN was found in *B. utilis* 22.50, 22.31 and 1.59 % respectively, while highest soil inorganic carbon (SIC) was found in the *P. smiothiana* followed by the *P. willachina* forest with a concentration of 0.43 and 0.24 %. Least STC, SOC and STN were found to be 0.87, 0.85 and 0.09 % in the mixed forest (PW, JE, BU) respectively, while least SIC was found to be 0.02 % in both the Mixed forest (PW, JE, BU) and Alpine grass each and 0.08 % in the *J. excelsa* forest (Fig. 6, Table 2).

**Table 2:** STN, SIC, SOC and STC under different forest types

Dominant trees	Total No. of plants	STN (%)	SIC (%)	SOC (%)	STC (%)
Mix.PW (224) + JE (31) + BU (18)	273	0.09	0.02	0.85	0.87
*Juniperus excelsa*	94	0.45	0.08	4.30	4.39
Mix.JE (42) + Pop. (32) + Astr(10)	84	0.49	0.07	4.79	4.86
Alpine grass	0	0.49	0.02	5.40	5.42
*Pinus wallichiana*	128	1.22	0.24	18.40	18.64
*Picea smithiana*	172	1.53	0.43	19.10	19.53
*Betula utilis*	128	1.59	0.19	22.31	22.50
Total	879	5.86	1.05	75.15	76.20

**Fig. 7:**
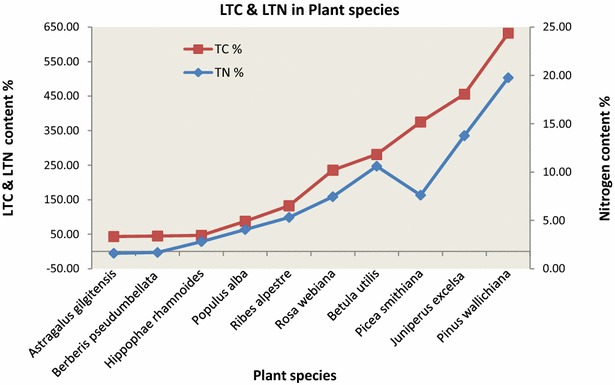
LTC and LTN in leaf samples of plant species

Leaf total carbon (LTC) concentration was highest in the leaves of *Pinus wallichiana* (*PW*); 632.54 followed by *J. excelsa*; 455.98 *Picea smithiana;* 375.45 *B. utilis;* 281.29 *Rosa webiana;* 235.64, *Ribes alpestre*; LTC concentration was lowest in the *Populus alba*.; 87.59, *Hippophae rhamnoides*; 46.62, *Berberis pseudumbellata;* 44.93 and *Astragalus gilgitensis*; 43.45. Leaf total nitrogen (LTN) concentration was highest in *P. wallichiana*; 19.77, followed by *J. excelsa*; 13.76, *B. utilis*; 10.61, *P. smithiana*; 7.62, *R. webiana*; 7.45, *R. alpestre*; 5.31 and *Populous alba* 4.06 and lowest in *H. rhamnoides*, 2.81, *B. pseudumbellata* 1.69 and *A. gilgitensis*; 1.60 (Fig. 7; Table 3).

**Table 3:** LTC and LTN in leaf samples of plant species

Plant species	LTN (%)	LTC (%)
*Astragalus gilgitensis*	1.60	43.45
*Berberis pseudumbellata*	1.69	44.93
*Hippophae rhamnoides*	2.81	46.62
*populous alba*	4.06	87.59
*Ribes alpestre*	5.31	132.48
*Rosa webiana*	7.45	235.64
*Betula utilis*	10.61	281.29
*Picea smithiana*	7.62	375.45
*Juniperus excelsa*	13.76	455.98
*Pinus wallichiana*	19.77	632.54

**Fig. 8:**
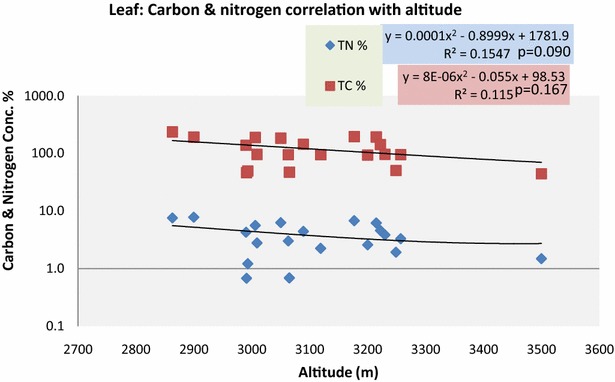
LTC and LTN correlation with altitude

LTC and LTN showed a weak correlation with altitude and did not differ significantly with R^2^ = 0.115, p = 0.167 for carbon and R^2^ = 0.154 p = 0.090 for nitrogen (Fig. 8). The mean results of T test for LTN and LTC in trees and shrubs were 3, 2.72 and 97.95, 74.23 and the results for all the parameters were found to be non-significantly different with p values 0.73 and 0.20 respectively (Table 4). The mean results of Paired T test for the LTN and LTC in the Conifers and deciduous plants were 2.26, 1.89 and 76.46, 46.36 and the results showed non-significantly different with p values such as 0.55 and 0.08 respectively (Table 5).

**Table 4:** T test for tree and shrub layer along the altitude

Altitude (m)	Tree (LTN)	Shrub (LTN)	Tree (LTC)	Shrub (LTC)
2863	4	3.6	147.1	89.5
2900	1.5	6.4	48.8	143.1
2990	2.5	1.8	94.6	44.2
3006	2.7	2.9	100.8	88.4
3009	1.2	1.6	52.6	43.4
3050	4.1	2.2	95.6	88.7
3089	2.6	1.9	100.5	44.4
3177	5.4	1.4	143.6	52.2
Total	24	21.8	783.6	593.9
Mean	3	2.725	97.95	74.2375
Variance	1.99	2.73	1286.19	1230.55
Observations	8	8	8	8
Hypothesized mean difference	0		0	
df	14		14	
t Stat	0.36		1.34	
p (T ≤ t) one-tail	0.36		0.10	
t critical one-tail	1.76		1.76	
p (T ≤ t) two-tail	0.73		0.20	
t critical two-tail	2.14		2.14

**Table 5:** Paired T test for conifers and deciduous trees along the altitude

Altitude (m)	Conifer (LTN)	Deciduous (LTN)	Conifer (LTC)	Deciduous (LTC)
3063	1.1	1.9	50.7	44.2
3177	3.2	2.2	100.2	43.4
3200	0.7	1.9	46.4	47.4
3215	3.9	2.3	145.1	48.3
3222	3.8	0.8	95.1	47.4
3230	1.6	2.3	48.7	47.5
3257	1.5	1.8	49	46.3
Total	15.8	13.2	535.2	324.5
Mean	2.26	1.89	76.46	46.36
Variance	1.79	0.27	1452.39	3.44
Observations	7	7	7	7
Pearson correlation	−0.23		0.18	
Hypothesized mean difference	0		0	
df	6		6	
t Stat	0.64		2.11	
p (T ≤ t) one-tail	0.27		0.04	
t critical one-tail	1.94		1.94	
p (T ≤ t) two-tail	0.55		0.08	
t critical two-tail	2.45		2.45	

**Fig. 9:**
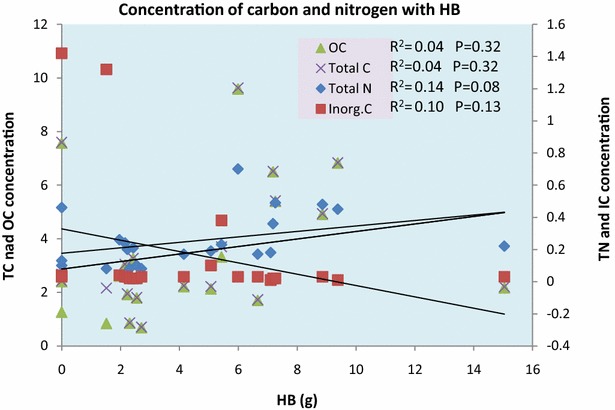
Correlation of SOC, STC, STN and SIC with HB

**Fig. 10:**
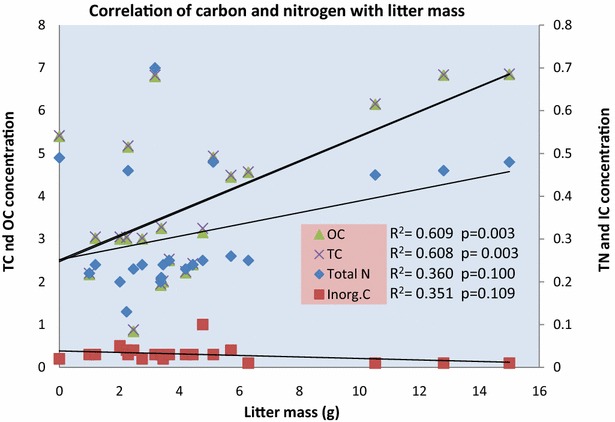
Correlation of SOC, STC, STN, SIC with LM

The correlation between the concentration of STN, STC and SIC with the HB along the altitudinal gradients was found to be weak non-significant with p values 0.085, 0.32 and 0.13 respectively. STC and SOC both showed a similar trend line and all were positive except HB with SIC (Fig. 9; Additional file 1: Annexure S2). The correlation of STC, STN and SIC with the LM along the altitudinal gradient was strongly significant positive, weak non-significant positive and weak non-significant negative with p values 0.003, 0.100 and 0.109 respectively (Fig. 10; Additional file 1: Annexure S2).

## Discussion

As the results show, with increasing altitude the STC, SOC and STN show increasing values but overall the altitudinal variation has little impact on soil carbon and nitrogen and the increasing variation are partly due to vegetation type and partly altitude. Both organic carbon and nitrogen show a strong positive correlation with the elevation and significantly differ. Our results support that the forest soil carbon density in Beijing also increases with increasing altitude (Xiao et al. 2014) and total organic N concentration significantly increased with elevation (Niklińska and Klimek 2007). The SIC showed the reverse results as with increasing altitude the inorganic carbon concentration decreased. The mineralization process increases in the organic regions with increasing elevation (Sveinbjörnsson et al. 1995) and soil respiration increases with increasing temperatures. Carbon and nitrogen content increased with increasing elevation and decreasing respiration rate due to decreasing temperature. The carbon and nitrogen stocks increased significantly with elevation as the soil and air temperature decreased with elevation (Vieira et al. 2011). Altitude is one of the factors influencing C pool in soil (Dar and Sundarapandian 2015). The results for the correlation of STC, SOC, SIC with the STN showed strongly positive significant, very strong positive significant and weak negative nonsignificant respectively. Our results support the findings of Knops and Timal (2000), that the rate of carbon accumulation depends on the rate of nitrogen accumulation and nitrogen accumulation depends on the biological fixation of nitrogen from atmosphere. At high elevations, the mineralization and decomposition processes are slow; as a result more nitrogen and carbon are found at high elevations as compared to lower elevations. It is found that N cycling is more sensitive to climate induced soil moisture variations, while C cycling is more strongly affected by temperature (Groffman et al. 2009). Our findings also show that nitrogen and carbon amount increases with increasing altitude.

Our findings showed clearly that SOC is the main contributor (75.1 %) to the carbon stock as compared to the SIC (1.05 %). Our results agree with the findings of Shi et al. (2012) in which they found SOC concentration was approximately nine times as high as SIC concentration. The averages of inorganic and organic carbon in the topsoil (0–20 cm) in grasslands of China were 0.38 and 3.63 % respectively. Many factors combined responsible for the SOC and SIC such as chemical and physical processes of soil formation drive the spatial pattern of SIC, while biotic processes and climatic factors drive the spatial pattern of SOC. SIC is controlled by soil acidification and other processes depending on soil pH. Vegetation type is the most important variable driving the spatial pattern of SOC (Shi et al. 2012). As this study was undertaken in a forest ecosystem. The forest soil is more humus as compared to other soils because of more organic carbon accumulation in the soil due to more litter and more decomposition process as compared to inorganic carbon. According to the tree models, carbonates and organic carbon in the topsoil were affected by different factors such as the pattern of SIC controlled by climate, soil physical and chemical properties (pH, Soil moisture etc.) while for SOC biotic and climatic factors were predominant. Vegetation type, AGB, BGB and LM are major drivers of the SOC (Shi et al. 2012). As a result of global changes, the temperature, precipitation, nitrogen availability has been altered (Rockström et al. 2009), these changes are most likely to have great impact on soil carbon. According to Wang et al. 2010) the density of organic carbon was highest in the forest and least in the desert while inorganic carbon had reverse results such as for SOC: Forest > grassland > shrub–grassland > shrub desert > desert; for SIC: forest, grassland < shrub–grassland < shrub desert < desert.

Soil organic matter (carbon and nitrogen) was highest in the broad leaved *B. utilis* forest followed by the *Picea smathiana*, *P. willachina* and lowest in the mixed forest (JE, Pop, Astr.) and Juniper forest. The broad leaved forest soil is rich with carbon and nitrogen contributing the most as a carbon sink in the area. The soil carbon is influenced by the vegetation type. Our results support the findings of Dar and Sundarapandian (2015) in which they also found highest carbon concentrations in the soil of BU forest at 65 %, significantly higher than those in other forests. Tree species are considered better indicators of soil carbon and nitrogen concentration than other factors (Vesterdal et al. 2002). Less carbon was found in the top mineral soil of deciduous forest floors. A study indicates that deciduous species have larger forest floor C pools as compared to other forest species (Oostra et al. 2006). Spruce trees have more carbon than beech in the western parts of Europe (Berger et al. 2002). The species types determine the nutrient cycling in the forest ecosystem. The canopy complexity in a mixed forest will have various different kinds of nutrients with different amounts. Similar heterogeneity of nutrients are produced by canopy complexity in the forest floor of mixed species. Soil nutrient production and availability depend on the mass of the litter and further depends on the canopy complexity of plant species. The effect of tree species can be better predicted from the mass and nutrient content of litter produced, hence total nutrient return, than from litter decay rate (Prescott 2002).

When all the plant leaf sample results were analyzed, it was found that LTC and LTN were highest in *Pinus wallichina* followed by *J. excelsa* then in *P. smithianaand* and *B. utilis*. Much smaller quantities were found in shrubs like *R. webiana*, *Hyphoe rhamnoides* etc. Among the shrubs LTC and LTN was highest in the *R. webiana* and least in the *A. gilgitensis*. Among the trees the least amount was found in the populous species which had a very small population size. In all other plant species like the shrubs the carbon and nitrogen was found to be very low in quantity. This indicates that in the study area PW species contributes the most as a LTC and LTN sink followed by JE species. The trees that are more numerous and larger in size contribute the most for soil organic matter as well as sinks for carbon in their leaves.

Vegetation type is the major cause of carbon and nitrogen concentration in the leaves, in our results there is a decreasing trend of carbon and nitrogen with increasing altitude. This is due to lower decomposition rates because of the decreasing temperature. The second major reason is the plant type as *P. wallichiana* leaves contain more carbon and nitrogen which is found at lower altitudes as compared to *B. utilis* which has lower carbon and nitrogen concentrations and is found at higher altitudes. Leaf to soil carbon and nitrogen show a weak positive correlation as leaves take nitrogen from the soil and carbon in the soil accumulates by root and microorganism respiration and decomposition of litter process. Leaf and soil carbon showed an increasing trend with increasing altitude except for the altitudes 2860 and 3500 m respectively. Leaf nitrogen also shows an increasing trend except at altitude 3500 m. As plant type is the more important factor when determining soil and leaf carbon and nitrogen, altitude so far does not have much impact except on the temperature which decreases at high elevations. With increasing altitude the temperature decreases which further decreases the mineralization rate. Our results, however, somehow support the findings of Zhang et al. (2012) that Soil N mineralization and nitrification rates decreased with increasing altitude. The variety and types of herbs and grasses like legumes cause more carbon and nitrogen accumulation in the soil. We examined the carbon and nitrogen concentration in the soil with increasing herbaceous biomass in each plot. As under the forest, very few herbs were found and a weak correlation with carbon and nitrogen was observed.

Our results support the findings that removal or addition of litter to the forest floor significantly affects the decrease and increase of the dissolved organic carbon and nitrogen (Park and Matzner 2003). More litter corresponds to more decomposition and as result more carbon and nitrogen production/accumulation in the soil. The other reason is that the N mineralization process and decomposition process varies from forest to forest depending on the quantity and quality of litter in the forest floor. Soil N and C are affected by anthropogenic and environmental changes which can be reduced by replacing species in the forest to retain N and carbon (Finzi et al. 1989). The live and dead parts of the coniferous and deciduous forest contained higher concentrations of soluble and total C and N and higher mineralization potentials than bare soil (Halvorson and Smith 2009). Litter is considered the most important factor when determining soil nutrient content. Nutrient production and availability depends largely on the litter. The decomposition of the litter and litter quality has great impact on the soil nutrient production and accumulation (Prescott 2002).

## Conclusion

STC, SOC and STN concentration showed a positive correlation, while SIC showed a negative correlation with increasing altitude. STC and SOC showed a strong positive relationship with STN while SIC showed a weak negative relationship with the STN and SOC. Broad leaved forests contribute the most to SOM as compared to mixed forests (PW, PS and JE). *P. wallichiana* leaves stock high carbon and nitrogen content as compared to the leaves of all other plant species. Shrubs stock less carbon as compared to trees. Altitude has a negligible positive impact on both LTC and LTN, but has a greater impact on shaping the vegetation structure which in further responsible for controlling the carbon and nitrogen concentration. LTC and LTN with respect to altitude and with respect to the forest type have positive correlations. HB had a weak correlation with STC and STN while LM contributes more to STN and STN. Mean TLC and TLN was higher in trees than in shrubs and in conifers than in deciduous plants. Forest type and LM both had a great impact on STC and STN, while altitude and HB had very little impact on these both. The carbon and nitrogen both show high impact on each other and are significantly correlated. Over all OC found to be the main contributor to both the TC stock and soil organic matter as compared to the IC and TN.

## Methods

### Study area

**Fig. 11:**
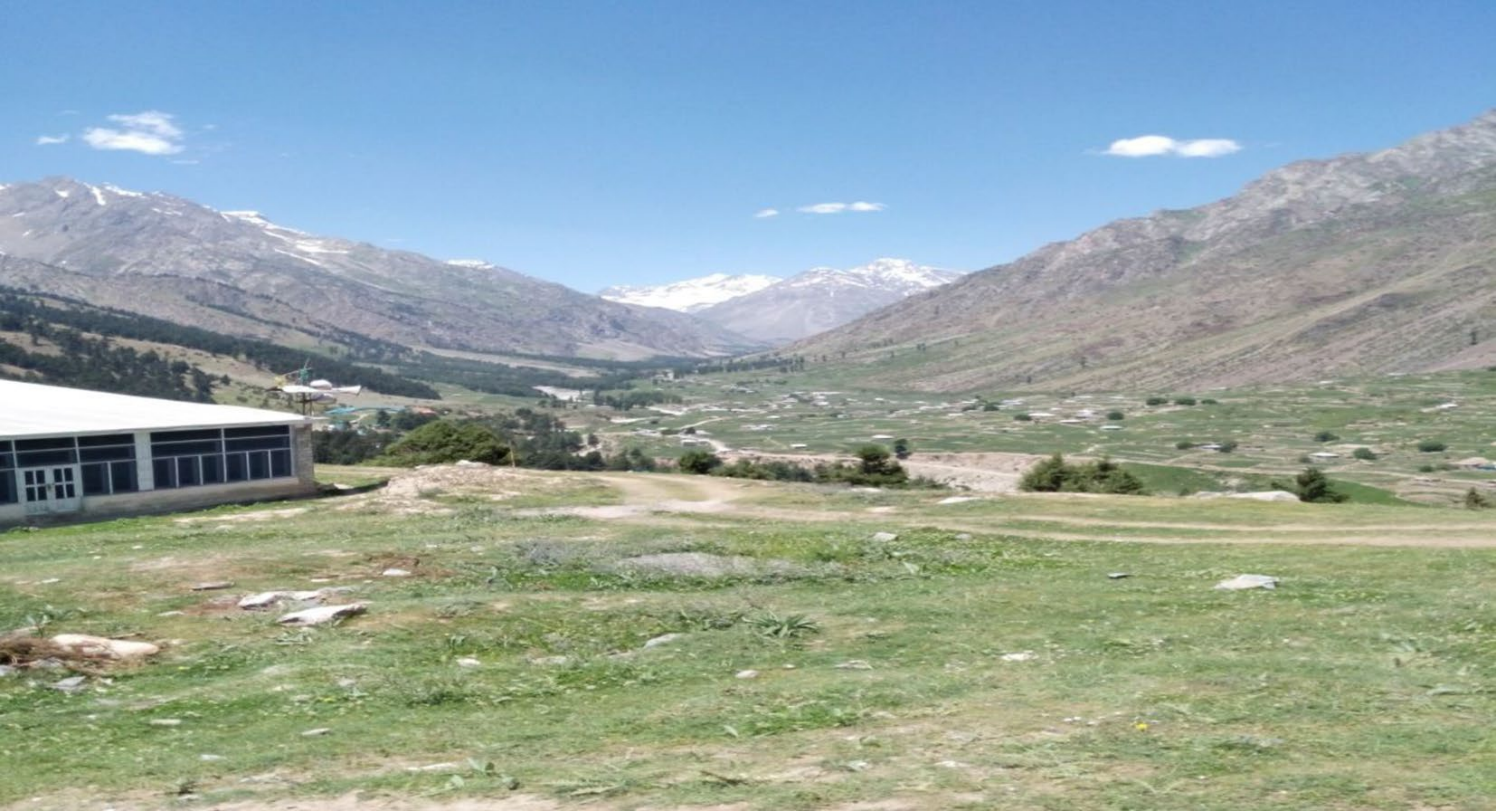
Image of the study area

Naltar Valley, a wildlife sanctuary, is located at N 36°09′42.2″ E 074°10′46.3″ and covers a total area of 27,206 ha (272.06 km^2^) in the Karakorum mountain ranges of Pakistan. The valley is designated as IUCN Management Category no. 4, according to IUCN-WCMC (1993). The altitude of the area ranges from 1700 to 5000 m (and above) ending in glaciers and the Naltar Pass. The dominant forest communities include *P. smithiana*, *P. wallichiana*, *J. excelsa*, and *B. utilis*. Other noticeable vegetation include Hippophae, Myricaria, Polygonum, Fragaria, Lonicera, Artemisia, Haloxylon and a variety of other species (Sheikh 1998). The upper and more open portion of the valley lies at 3000 m, where most of the human settlements and activities are found (IUCN-WCMC 1993). Glaciations have caused the formation of moraines due to the continuous geographical changes. In addition to natural geographic changes, man-made changes have also been introduced to the valley’s landscape, such as the expansion of agriculture, which has increased due to the cutting of forests in the lower reaches of the valley and the valley basin (Fig. 11). The winter is very harsh, with temperatures falling below −18 °C, and an annual rainfall of 254–400 mm (Sheikh et al. 2002).

### Data collection and process

Field visits were conducted from June to August 2014 to collect soil and leaf samples from 22 different points with increasing altitude and vegetation types. The coordinates and elevation was recorded with the help of GPS. In each stand a 20 m^2^ circular plot was laid to collect leaf samples of the tree species. Within the 20 m circular plot 5 m^2^ plots was laid to get leave/branch samples of the shrubs. Four 1 m^2^ rectangular plots were laid down to obtain herb and grass samples. Within each 1 m^2^ plot a small 0.25 m^2^ plot was laid. The fresh weight of the individual herbaceous roots and shoots were noted with the help of an electric balance. Fresh weight of litter was taken from a small 0.25 m^2^ plot laid in the centre of each 20 m^2^ circular plot. From each plot soil samples were collected from a 25 cm depth with the help of a soil auger. All leaf, litter and soil samples were packed in tagged bags and then transported to the lab for dry weight and chemical analysis. The leaves were oven dried while soil samples were air dried. The leaf samples were then ground to powder (2 mm) in a Retsch machine and soil samples were also sieved to a size of 2 mm. Total carbon, organic carbon and total nitrogen was analysed by dry method using All of the samples were then analyzed by the Dry method using an Elemental analyzer (Model: vario macro cube, Germany) for total carbon, organic carbon and total nitrogen content while inorganic carbon was analyzed by Gas law using a Carbonate analyzer (Eijkelkamp 08.53). The results were then analyzed for correlation by a linear regression model and the Pearson’s correlation coefficient r with the computational formula and their significance level p was determined to find the relationship status and significance level. The comparisons between trees and shrubs and between conifers and deciduous plants were performed by paired T test of two samples for means and T test assuming unequal variances respectively.

## Additional file

10.1186/s40064-016-1935-9 Total carbon and total nitrogen correlation with altitude, HB and LM.

## Abbreviations

STC: soil total carbon
SOC: soil organic carbon
SIC: soil inorganic carbon
STN: soil total nitrogen
HB: herbaceous biomass
HAGB: herbaceous above ground biomass
HBGB: herbaceous below ground biomass
LM: litter mass
LTC: leaf total carbon
LTN: leaf total nitrogen
PW: *Pinus wallichina*
PS: *Picea smathina*
JE: *Juniperus excelsa*
BU: *Betula utilis*
SOM: soil organic matter
WWF: World Wide Fund for Nature
IUCN: International Union for Conservation of Nature
WCMC: World Conservation and Monitoring Centre
