# Supplementary material for: Altitudinal gradients of soil and vegetation carbon and nitrogen in a high altitude nature reserve of Karakoram ranges
Source: Springerplus. 2016 Mar 12;5:320. doi: 10.1186/s40064-016-1935-9 (PMC4788676; doi:10.1186/s40064-016-1935-9)
Supplement: Supplementary file 1 — 10.1186/s40064-016-1935-9 Total carbon and total nitrogen correlation with altitude, HB and LM. [file 40064_2016_1935_MOESM1_ESM.docx]

**Additional files**

**Annexure S1:** LTC and LTN correlation with altitude

| **Altitude (m)** | **LTN %** | **LTC %** |
| --- | --- | --- |
| 2863 | 7.6 | 236.6 |
| 2900 | 7.8 | 191.8 |
| 2990 | 4.3 | 138.8 |
| 2991 | 0.7 | 46.4 |
| 2993 | 1.2 | 49.5 |
| 3006 | 5.6 | 189.2 |
| 3009 | 2.8 | 96.0 |
| 3050 | 6.3 | 184.3 |
| 3063 | 3.0 | 94.9 |
| 3065 | 0.7 | 47.1 |
| 3089 | 4.4 | 144.9 |
| 3119 | 2.2 | 95.2 |
| 3177 | 6.8 | 195.9 |
| 3200 | 2.6 | 93.8 |
| 3215 | 6.2 | 193.4 |
| 3222 | 4.6 | 142.5 |
| 3230 | 3.9 | 96.2 |
| 3249 | 1.9 | 50.6 |
| 3257 | 3.3 | 95.3 |
| 3500 | 1.5 | 44.3 |
|  |  |  |

**Annexure S2:** STN, SIC, SOC and STC concentration with respect to HB and LM

| **HB (AGB+BGB)** | **STN** | **SIC** | **SOC** | **STC** | **LM** | **STN** | **SIC** | **SOC** | **STC** |
| --- | --- | --- | --- | --- | --- | --- | --- | --- | --- |
| 1.52 | 0.23 | 0.04 | 0.84 | 0.88 | 1 | 0.2 | 0.03 | 2.17 | 2.2 |
| 1.96 | 0.26 | 0.04 | 2.61 | 2.65 | 1.2 | 0.2 | 0.03 | 3.02 | 3.05 |
| 2.15 | 0.24 | 0.03 | 3.02 | 3.05 | 2 | 0.2 | 0.05 | 3 | 3.05 |
| 2.23 | 0.2 | 0.03 | 1.92 | 1.95 | 2.2 | 0.1 | 0.04 | 3 | 3.04 |
| 2.31 | 0.08 | 0.02 | 0.85 | 0.87 | 2.3 | 0.5 | 0.03 | 5.15 | 5.18 |
| 2.43 | 0.21 | 0.02 | 3.25 | 3.27 | 2.5 | 0.2 | 0.04 | 0.84 | 0.88 |
| 2.55 | 0.1 | 0.02 | 1.79 | 1.81 | 2.8 | 0.2 | 0.02 | 3 | 3.02 |
| 2.71 | 0.08 | 0.03 | 0.68 | 0.71 | 3.2 | 0.7 | 0.03 | 9.59 | 9.62 |
| 4.15 | 0.17 | 0.03 | 2.21 | 2.25 | 3.4 | 0.2 | 0.03 | 1.92 | 1.95 |
| 5.07 | 0.19 | 0.1 | 2.13 | 2.22 | 3.4 | 0.2 | 0.02 | 3.25 | 3.27 |
| 5.43 | 0.23 | 0.38 | 3.32 | 3.7 | 3.5 | 0.2 | 0.02 | 2 | 2.02 |
| 5.99 | 0.7 | 0.03 | 9.59 | 9.63 | 3.7 | 0.3 | 0.03 | 2.5 | 2.53 |
| 6.66 | 0.17 | 0.03 | 1.7 | 1.73 | 4.2 | 0.2 | 0.03 | 2.21 | 2.24 |
| 7.1 | 0.18 | 0.01 | 2.46 | 2.48 | 4.5 | 0.2 | 0.03 | 2.4 | 2.43 |
| 7.18 | 0.36 | 0.02 | 6.5 | 6.52 | 4.8 | 0.3 | 0.1 | 3.15 | 3.25 |
| 7.26 | 0.49 | 0.02 | 5.4 | 5.42 | 5.1 | 0.5 | 0.03 | 4.91 | 4.94 |
| 8.86 | 0.48 | 0.03 | 4.91 | 4.94 | 5.7 | 0.3 | 0.04 | 4.45 | 4.49 |
| 9.38 | 0.45 | 0.01 | 6.83 | 6.84 | 6.3 | 0.3 | 0.01 | 4.56 | 4.57 |
| 15.04 | 0.22 | 0.03 | 2.17 | 2.21 | 11 | 0.5 | 0.01 | 6.15 | 6.16 |
| 99.98 | 5.04 | 0.92 | 62.18 | 63.13 | 13 | 0.5 | 0.01 | 6.83 | 6.84 |
|  |  |  |  |  | 15 | 0.5 | 0.01 | 6.85 | 6.86 |
|  |  |  |  |  | 100 | 6.4 | 0.64 | 80.95 | 81.59 |
